# Rapid Discrimination of Methicillin-Resistant *Staphylococcus aureus* by MALDI-TOF MS

**DOI:** 10.3390/pathogens8040214

**Published:** 2019-11-01

**Authors:** Jung-Min Kim, Inhee Kim, Sung Hee Chung, Yousun Chung, Minje Han, Jae-Seok Kim

**Affiliations:** 1 Department of Laboratory Medicine, Hallym University College of Medicine, Seoul 05355, Korea; jungmin510@gmail.com (J.-M.K.); inhee6389@gmail.com (I.K.); ssung929@gmail.com (S.H.C.); 2 Department of Laboratory Medicine, Kangdong Sacred Heart Hospital, Hallym University College of Medicine, Seoul 05355, Korea; yousun623@gmail.com (Y.C.); minjehan@gmail.com (M.H.)

**Keywords:** *Staphylococcus aureus*, MRSA discrimination, MALDI-TOF MS

## Abstract

Methicillin-resistant *Staphylococcus aureus* (MRSA) is a serious pathogen in clinical settings and early detection is critical. Here, we investigated the MRSA discrimination potential of matrix-assisted laser desorption ionization time-of-flight mass spectrometry (MALDI-TOF MS) using 320 clinical *S. aureus* isolates obtained in 2005–2014 and 181 isolates obtained in 2018. We conducted polymerase chain reactions (PCR) for staphylococcal cassette chromosome *mec* (SCC*mec*) typing and MALDI-TOF MS to find specific markers for methicillin resistance. We identified 21 peaks with significant differences between MRSA and methicillin-susceptible *S. aureus* (MSSA), as determined by *mecA* and SCC*mec* types. Each specific peak was sufficient to discriminate MRSA. We developed two methods for simple discrimination according to these peaks. First, a decision tree for MRSA based on six MRSA-specific peaks, three MSSA-specific peaks, and two SCC*mec* type IV peaks showed a sensitivity of 96.5%. Second, simple discrimination based on four MRSA-specific peaks and one MSSA peak had a maximum sensitivity of 88.3%. The decision tree applied to 181 *S. aureus* isolates from 2018 had a sensitivity of 87.6%. In conclusion, we used specific peaks to develop sensitive MRSA identification methods. This rapid and easy MALDI-TOF MS approach can improve patient management.

## 1. Introduction

*Staphylococcus aureus* is a major human pathogen that causes various diseases, including food poisoning, toxic shock syndrome, abscess, pneumonia, and sepsis [1]. Resistance to methicillin is caused by the *mecA* gene, which encodes an alternative penicillin-binding protein (PBP2a). Methicillin-resistance of *S. aureus* is attributed to the insertion of the staphylococcal cassette chromosome *mec* (SCC*mec*), a mobile genetic element, carrying the *mecA* into the chromosome of susceptible strains [2], with the SCC*mec* type reflecting the clonality of *S. aureus* [3]. Since the initial spread of methicillin-resistant *S. aureus* (MRSA) in hospital settings in the late 1980s, the infection rate has increased sharply, account for a huge number of deaths worldwide [4,5]. For proper antibiotic treatment, rapid discrimination between MRSA and methicillin-susceptible *S. aureus* (MSSA) is required.

Culture-based antimicrobial susceptibility testing, a standard method for the detection of methicillin resistance in clinical laboratories, is time-consuming [6]. Several alternative MRSA detection methods have been developed, such as selective chromogenic media, PCR assays, and, more recently, the matrix-assisted laser desorption ionization time-of-flight mass spectrometry (MALDI-TOF MS) [6,7,8,9]. Detection of MRSA is divided into two general type of techniques such as the performance/efficacy criteria or convenience/efficiency criteria. The polymerase chain reaction (PCR)-based methods have the advantages of high performance and efficacy, and qPCR or RT-qPCR, ddPCR, and modified 16S sequencing require only a few hours [10,11]. In contrast, the MALDI-TOF MS, has the potential to become a convenient and efficient type of detection method for MRSA identification, as it is already routinely used in many clinical microbiology laboratories; indeed, The detection of antibiotic resistance by MALDI-TOF MS is receiving substantial attention [12]. This technique can be performed in a few minutes with a single colony, costs only a few US dollars, and there is no additional cost if a laboratory already has a MALDI-TOF MS setup for bacterial identification. MALDI-TOF MS could be used to identify bacterial genera and species as well as intraspecific properties, such as clonal complexes and/or spa types of *S. aureus* [13,14,15,16]. Since susceptibility testing by MADI-TOF MS was first reported in 2000 [6], MRSA spectra have been evaluated extensively, including analyses of single peaks, clusters, or whole spectra [17,18,19]. The PBP2a has a direct effect on methicillin resistance, but cannot be detected by MALDI-TOF MS owing to its molecular weight of 76 kDa [20]. Some researchers have used MRSA-related special peaks, such as PSM-mec, which is related to methicillin resistance [19,21,22,23,24]. Several peaks can powerfully discriminate some MRSA, but may not be sufficient to detect all MRSA. Thus, this study aimed to identify specific markers for discrimination between MRSA and MSSA by MALDI-TOF MS, and to develop discrimination methods that can be applied to clinical *S. aureus* isolates. 

## 2. Results

### 2.1. Specific Peaks for MRSA and MSSA

Methicillin resistance was evaluated by multiplex PCR (M-PCR) for *mecA* gene and SCC*mec* type. 

We used two sets of *S. aureus* as the database set and test set. The database set comprised of 320 *S. aureus* isolated during 10 years, while the test set contained 181 recently isolated *S. aureus*. Among the 320 isolates of the database set, 213 were MRSA and 107 were MSSA. Among the 213 MRSA isolates, 72.8% were SCC*mec* type II (155 cases), 11.7% were type III (25 cases), and 15.5% were type IV (33 cases). All 320 *S. aureus* isolates were identified by MALDI-TOF MS at the species level with a score value of >2.0. After a spectral analysis, we obtained 21 peaks by MALDI-TOF MS that differed significantly with respect to *mecA* or SCC*mec* type (*P* < 0.01). Thirteen peaks were identified as MRSA-specific markers. Among them, peaks at m/z 2204, 2410, 2592, 4607, and 9216 were predominantly detected in MRSA strains and in some MSSA isolates (Table 1). Eight peaks were MSSA-specific markers and peaks at m/z 2339, 3034, and 5509 had specificities of greater than 91% (Table 1). Distinguishing MSSA and MRSA strains can serve as an effective transition to further characterize MRSA strains and test whether the MALDI-TOF method can distinguish between those strains.

### 2.2. Specific Peaks According to MRSA SCCmec Type

**Figure 1:**
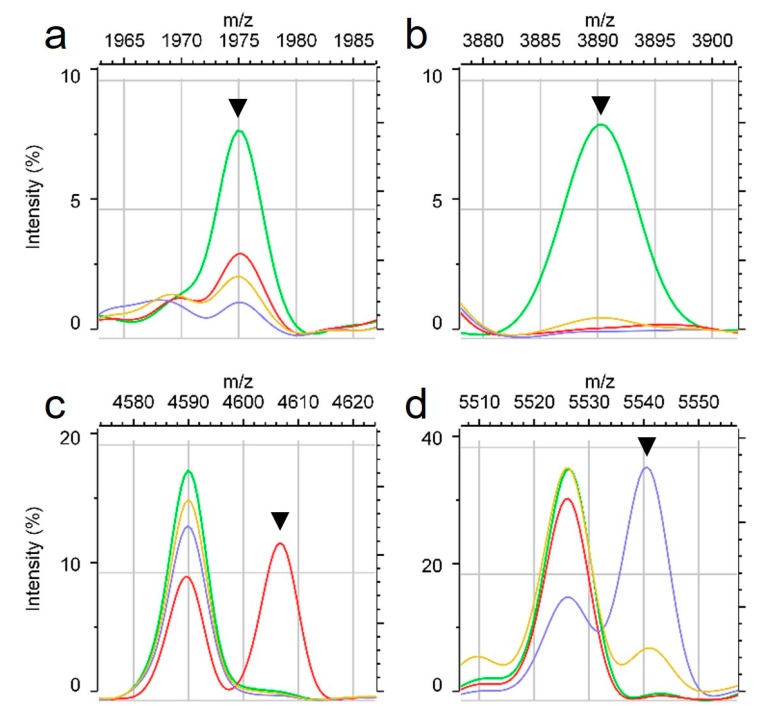
Average spectra for *S. aureus* according to SCC*mec* type. Light green line indicates MRSA SCC*mec* type II, red line is type III, blue line is type IV, and yellow line is MSSA. Peaks are indicated by arrows specific to an SCC*mec* type, including a type II-specific peak at m/z 1975 (**a**) and m/z 3890 (**b**); type III-specific peak at m/z 4607 (**c**); type IV-specific peak at m/z 5541 (**d**).

We detected peaks with different characteristics depending on SCC*mec* type. Peaks at m/z 1975, 2592, and 3890 were specific to SCC*mec* type II and had specificities and positive-predictive values of greater than 80% (Table 1, Figure 1a,b). The peaks at m/z 2204, 2410, 4607, 6594, and 9216 were specific to SCC*mec* type III. In particular, 100% of SCC*mec* type III carriers exhibited peaks at m/z 2410, 4607 (Figure 1c), and 9216. The PSM-mec peak was detected between m/z 2407 and 2412 (median, m/z 2410). For SCC*mec* type IV prediction, we selected three peaks. However, MALDI spectra for SCC*mec* type IV strains were similar to those for MSSA strains, with overlapping prediction peaks. Similarly, most MSSA prediction peaks were found in some SCC*mec* type IV isolates, but peaks at m/z 5541 and 5579 were more frequently observed in SCC*mec* type IV than in MSSA isolates (Table 1 and Figure 1d). Peaks at m/z 5541 and 5579 were detected in greater than 90% of SCC*mec* type IV isolates. 

### 2.3. Decision Tree for MRSA and MSSA

**Figure 2:**
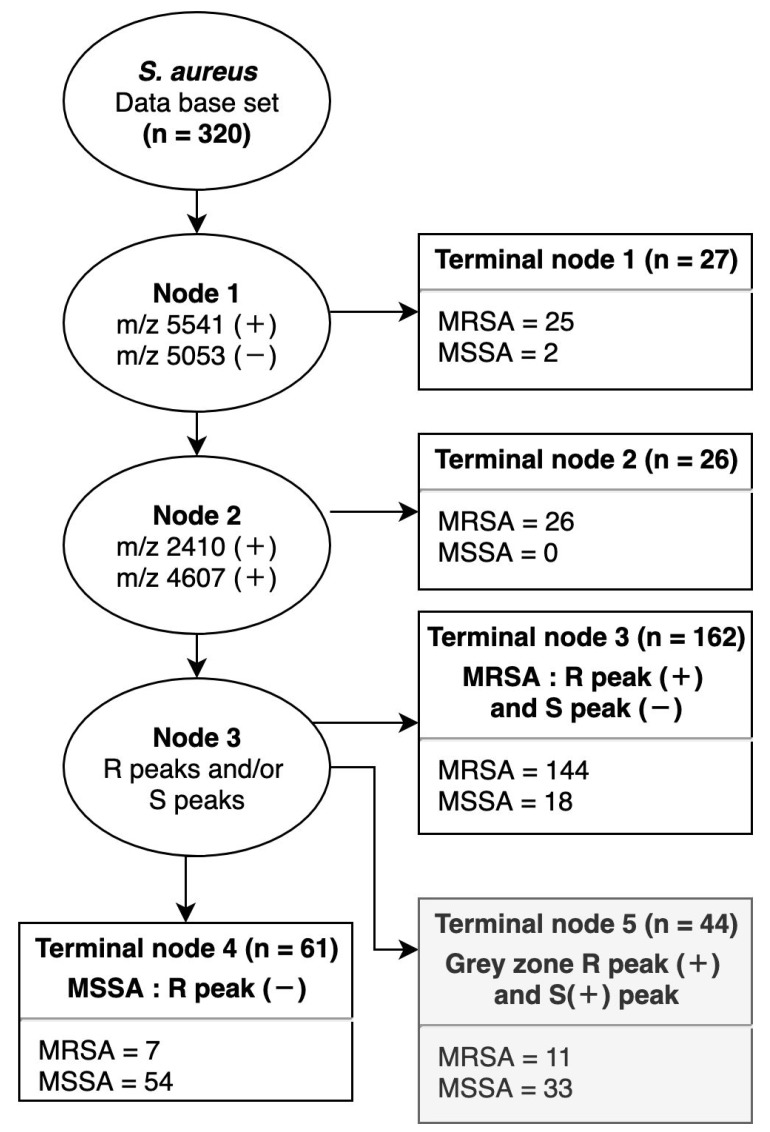
Decision tree for MRSA and MSSA. Decision tree for MRSA and MSSA applied to the database set. At node 1, peaks at m/z 5541 and 5053 were used for SCC*mec* type IV classification. At node 2, peaks at m/z 2410 and 4607 were used for SCC*mec* type III classification. At node 3–5, R peaks (MRSA prediction peaks at m/z 1975, 2410, 2592, 3890, 4607, and 6594) and S peaks (MSSA prediction peaks at m/z 2194, 2339, and 2631) were used. Terminal node 3 identifies MRSA that expressed at least one or more R peaks and no S peaks. Terminal node 4 identifies MSSA with no expression of R peaks. Terminal node 5 determines unclassifiable isolates that express at least one or more R peaks and S peaks (referred to as the grey zone).

To identify MRSA by specific peaks, we made a decision tree with three nodes containing 11 peaks with high sensitivity and specificity (Figure 2). First, node 1 was used for the classification of SCC*mec* type IV based on the absence of the peak at m/z 5053 and the presence of the peak at m/z 5541. A total of 320 spectra were classified using node 1 into 25 MRSA and 2 MSSA. All 25 MRSA were SCC*mec* type IV strains. The remaining 293 spectra were classified using node 2 based on m/z 2410 and 4607, specific for SCC*mec* type III. Node 2 classified 26 MRSA, including one strain of type II and 25 strains of type III. Then, 267 spectra were classified as MRSA or MSSA (node 3). If at least one out of six peaks for MRSA prediction was present (m/z 1975, 2410, 3890, 4607, and 6594) and no peak for MSSA prediction was present (m/z 2194, 2339, and 2631), the isolate was classified as MRSA (terminal node 3). Spectra with no peak for MRSA prediction were classified as MSSA (terminal node 4). If peaks for both MRSA and MSSA were detected, the isolate was unclassified (i.e., assigned to a grey zone; terminal node 5). By node 3, 144 MRSA and 54 MSSA were classified. According to this decision tree, 96.5% of MRSA and 73.0% of MSSA were finally identified. Including a decision tree for a benchmarked method (e.g., the multiplexed PCR at the beginning of Section 2.1) will add to the validation of this assay by demonstrating that the MALDI-TOF method performs as well as a conventional test.

### 2.4. Simple Prediction Model Based on a Combination of Specific Peaks

To simplify prediction, we generated combinations of highly specific peaks for MRSA and MSSA. MRSA-specific peaks were selected at m/z 1975, 2410, 3890, and 5541, and MSSA-specific peaks were selected at m/z 2194, 2230, and 2339. Four-peak combinations (Table 2) showed improved MRSA prediction ability compared with that for single peaks (PSM-mec, 46%). Using only the combination of MRSA-specific peaks, the MSSA sensitivity was low, i.e., 45.8%. We added one MSSA-specific peak, but the difference in sensitivity was not substantial. The combination with the peak at m/z 2339 showed high predictive value for MRSA, with 88.3% sensitivity for MRSA and 67.3% for MSSA. Therefore, this simple method of discrimination is more sensitive than the one-peak method, but less sensitive than the decision tree.

### 2.5. Evaluation of the Test Set

A total of 181 *S. aureus* collected in 2018 were used as the test set for evaluating the decision tree and simple discrimination method. The decision tree showed sensitivities of 87.6% for MRSA and 74.4% for MSSA (Table 3). Sensitivities for simple discrimination based on the 4 MRSA specific peaks and m/z 2339 peak were 67.4% for MRSA and 58.1% for MSSA, which were lower than those for the database set. Increasing MSSA sensitivity by simple determination could not be achieved.

## 3. Discussion

This study demonstrates the ability of the MALDI-TOF MS method to efficiently discriminate MRSA and MSSA strains through specific novel peaks that were identified. Previous studies have also identified specific peaks for MRSA, but a routine discrimination method for clinical settings based on these peaks is still lacking. Moreover, the reported peaks exhibit different patterns [14,25] or are applicable to only some MRSA strains [17,23,27]. In contrast, the discrimination methods described herein show high accuracy and good predictive value, and have the scope to be applied in routine clinical practice.

Previously, a PSM-mec peak at m/z 2415 ± 4 was reported as a prediction marker for MRSA [19,23]. In the current study, a PSM-mec peak was found at m/z 2410 ± 3 and appeared in the spectra for 46% of MRSA. The PSM-mec peak has the highest predictive value using the single peak prediction method. Other studies have reported a MRSA prediction peak at m/z 3890, which was also observed in our study [13,24]. In our MRSA isolates, this m/z 3890 peak was specific for SCC*mec* type II and did not appear in the spectra for type III or type IV isolates. These two peaks could identify MRSA, but showed a sensitivity of only 40–46%. 

Peak shifts for *S. aureus* can be explained by different clonal groups or mutations [13,24,26]. The peak at m/z 6594 has been reported as a shift from m/z 6552, similar to peaks at m/z 6580 and 6568 [28]. In our study, most strains showed the original peak at m/z 6552 (m/z 6554 in this study), but 92% of SCC*mec* type III strains showed characteristic peak shifts to m/z 6594 (Table 1). This peak at m/z 6594 was specific to SCC*mec* type III. Additionally, peaks at m/z 4607 and 9216 were specific markers for MRSA, especially SCC*mec* type III that have 100% sensitivity (Table 1 and Figure 1c).

*S. aureus* SCC*mec* type IV is a community-acquired MRSA (CA-MRSA) and is genetically and phenotypically distinct from healthcare-associated MRSA (HA-MRSA). Infections with SCC*mec* type IV are increasing and causing diseases of the skin and soft tissue. CA-MRSA tends to be susceptible to most antibiotics other than methicillin and beta-lactamase [29]. Therefore, the accurate discrimination of not only MRSA but SCC*mec* types can enable the selection of a variety of effective antibiotics. In our experiment, it was not easy to discriminate between SCC*mec* type IV and MSSA owing to similar spectral patterns. The peak at m/z 5525 has three different shift forms at m/z 5507, 5551, and 5539 [30]. In this study, the original peak at m/z 5525 was found in 81.6% of *S. aureus.* The shifted peak at m/z 5507 (m/z 5509 in our study) was found in 20% of MSSA isolates and appeared to be MSSA-specific. The peak at m/z 5539 (m/z 5541 in this study) was found in most SCC*mec* type IV isolates. In particular, this peak was detected in 76% of SCC*mec* type IV isolates (Figure 1d) and 24% of MSSA isolates. The shifted peak at m/z 5541 was specific to SCC*mec* type IV, although it had a low positive predictive value (Table 1). We used this peak in node 1 of the decision tree for MRSA discrimination (Figure 2). Additionally, we used the peak at m/z 5053 in node 1 of the decision tree owing to its high positive predictive value of 98% for all isolates, except SCC*mec* type IV. As a result, we could discriminate MRAS SCC*mec* type IV more accurately by reducing similarity with spectral patterns for MSSA. These SCC*mec* type IV-specific peaks could be used as a basis for antibiotic therapy because type IV strains are susceptible to various antibiotics [31,32].

We found dense MRSA-specific peaks between m/z 2000–4000. Typically, MALDI-TOF MS spectra are analyzed in the range of m/z 2000–20000 after processing steps, such as baseline subtraction and peak selection. However, we found a specific peak at m/z 1975 before limiting the *x*-axis dimensions (Figure 1a). In our study, this peak was found in spectra for numerous MRSA isolates. The PSM-mec peak was found in 46% of MRSA isolates, but the peak at m/z 1975 was found in 61% of MRSA isolates. Though this peak was found in some MSSA, the peak at m/z 1975 can detect several strains that cannot be identified by the PSM-mec peak. Therefore, the peak at m/z 1975 can be broadly applied for the discrimination of MRSA. This result also demonstrated the potential for MRSA-specific peaks with low masses (i.e., under m/z 2000), emphasizing the importance of studying the low mass area.

Some of the specific peaks discovered in our analysis have been described previously, with the same or different interpretation. The use of MALDI-TOF MS to identify MRSA from unidentified strains has been controversial. Some studies have reported an inability to find a distinctive fingerprint for MRSA [11,17,33,34]. MALDI-TOF MS does not consistently yield the same intensities or peak patterns; therefore, various database of specific peaks for MRSA is needed.

We tested reference strains, including ATCC 43300, ATCC 29213, and RN4220 (Supplementary Figure S1 and Table S1). Unfortunately, MRSA ATCC 43300 and RN4220 strain could not be classified, as they did not express any of the peaks among the decision tree containing 11 peaks. The MSSA ATCC 29213 was classified to the grey zone, because this strain expressed specific peaks of both MRSA and MSSA. These reference strains were collected many years ago; therefore, the MALDI-TOF peaks may not reflect the protein expression profiles of recent clinical isolates. Nonetheless, additional analyses, such as spa typing or MLST analyses, are needed for the accurate detection of various types of *S. aureus* isolates from Korea as well as from other countries. Discovering specific peaks for each clonal type will increase accuracy. 

We categorized peaks only by SCC*mec* type for easy and rapid classification. The specificity of some peaks reported in our study has already been described for each MLST type [14]. Previous studies have shown that the PSM-mec peak is specific to MRSA ST239 in Taiwan and the peak at m/z 3890 is specific to MRSA ST5 [24]; however, another study has reported that this latter peak has specificity for MSSA ATCC 29213 [13]. In our study, this peak was detected in 55% of SCC*mec* type II isolates. The peak at m/z 5509 was reported to be specific to CC30 of MRSA [30]. However, this peak was specific to MSSA in our study. The peak at m/z 6594 was previously reported to be specific to CC8, CC30, and ST239 of MRSA in Europe [24,28,30]; it was specific to MRSA SCC*mec* type III in our study.

Several studies have attempted to discriminate MRSA by single peaks or peak clustering. However, the sensitivity of PSM-mec peaks is only 39% [23], and that of two peaks in combination is only 63% [27]. Accordingly, discrimination based on only a few peaks is not highly accurate. Therefore, we constructed a decision tree using peaks with features associated with SC*Cmec* type. 

In conclusion, the 21 peaks and two discrimination methods described in our study provide exceptional predictive value, as high as 96.5%. These peaks can be used as basis for the accurate classification of MRSA. Using 181 clinical *S. aureus* isolates collected in 2018, the decision tree enabled the accurate identification of 87.6% of isolates. Thus, this approach is powerful for the discrimination of MRSA. MALDI-TOF, which can be used to identify strains and species, can also be used to distinguish between MRSA and MSSA. Therefore, the newly developed prediction methods will facilitate accurate and rapid diagnosis.

## 4. Materials and Methods 

### 4.1. Bacterial Strains

Two sets of *S. aureus* clinical isolates, a database set and test set, were included. The database set included 320 *S. aureus* isolates that were randomly collected at Hallym University Kangdong Sacred Heart Hospital, Seoul, Korea from 2005 to 2014 (approximately 30 strains per year). The test set consisted of 181 isolates that were randomly selected in 2018 for the evaluation of the MRSA identification scheme. *S. aureus* was cultivated on blood agar plates for 18 h at 37 °C and 5% CO_2_. 

### 4.2. MALDI-TOF Measurements of Bacterial Cells

Mass spectra were collected using a Microflex LT MALDI-TOF MS instrument (Bruker Daltonics, Bremen, Germany) operated by FlexControl v3.4. A bacterial colony was directly smeared on a steel MALDI plate without protein extraction. Dried samples were overlaid with 1 μl of formic acid and 1 μl of CHCA matrix solution (saturated solution of α-cyano-4-hydroxycinnamic acid in 50% acetonitrile with 2.5% trifluoroacetic acid), sequentially. The Bruker Bacterial Test Standard was used for mass calibration. The acquired raw spectra were exported from FlexAnalysis (Bruker Daltonics). Spectra were obtained in the range of 1960 to 20000 m/z. 

### 4.3. Screening for the mecA Gene and SCCmec Type

Chromosomal DNA was extracted from *S. aureus* using the HiYield Genomic DNA Mini Kit (Real Biotech Corporation, Banqiao City, Taiwan) according to the manufacturer’s instructions. The *mecA* gene and SCC*mec* type were evaluated by two steps of M-PCR using the QIAGEN Multiplex PCR Master Mix (Qiagen, Hilden, Germany) and SCC*mec* element type primers [35]. M-PCR step 1 identified *mecA* and the *ccr* region. M-PCR step 2 identified the gene lineages of *mecA*-*mec*I, *mecA*-IS1272, and *mecA*-IS431.

### 4.4. Analysis of Mass Spectra

MALDI-TOF spectra were preprocessed using BioNumerics v7.6 (Applied Maths, Belgium) by recalibration, baseline subtraction (Top), and peak selection. Peak lists generated by BioNumerics software were analyzed using SPSS version 24. Correlations between peaks and *mecA* were evaluated by Pearson’s chi-square tests. A *P*-value of <0.05 indicated statistical significance.

### 4.5. Ethical Approval

The manuscript contains no data concerning animal studies, studies involving human subjects or inclusion of identifiable human data or clinical trials; thus, no ethical approval was required.

## Figures and Tables

**Table 1:** Specific peaks for the discrimination of *S. aureus* SCC*mec* type.

Type	Peak (m/z)	SCC*mec*			MSSA	SE	SP	PPV	NPV	*P*	Decision Tree	Reference
		II	III	IV								
MRSA SCC*mec* type II	1975	81	12	6	26	80.6	80.0	79.1	81.5	<0.001	node 3	
	2134	65	52	27	30	65.2	67.3	65.2	67.3	<0.001		
	2592	24	12	3	4	23.9	95.2	82.2	57.1	<0.001	node 3	
	3890	55	0	0	3	54.8	98.2	96.6	69.8	<0.001	node 3	[9] *, [10] ^†^, [12] ^†^, [20] ^†^
MRSA SCC*mec* type III	2204	3	68	0	0	68.0	98.3	77.3	97.3	0.001		
	2410	43	100	18	15	100.0	69.8	21.9	100.0	<0.001	node 2, 3	[15] ^†^, [17,18,19,20] ^†^
	2874	6	64	9	5	64.0	94.2	48.5	96.9	<0.001		
	4607	1	100	0	0	100.0	99.7	96.2	100.0	<0.001	node 2, 3	
	6594	3	92	9	12	92.0	93.2	53.5	99.3	<0.001		[12] ^†^, [20] ^†^, [25] ^※^, [26] ^†^
	9216	1	100	0	0	100.0	99.7	96.2	100.0	<0.001		
MRSA SCC*mec* type IV	5053	98	80	21	91	N/A				<0.001	node 1	
	5541	1	0	76	24	75.8	90.2	47.2	97.0	<0.001	node 1	[10] ^†^
	5579	0	0	70	11	69.7	95.8	65.7	96.5	<0.001		
MSSA	2194	2	24	76	55	55.1	84.0	63.4	78.9	<0.001	node 3	
	2232	1	8	55	37	37.4	90.1	65.6	74.1	<0.001	node 3	
	2301	6	56	94	74	73.8	74.6	59.4	85.0	<0.001		
	2339	1	0	52	34	33.6	91.5	66.7	73.3	<0.001		
	2631	5	44	85	66	66.4	77.9	60.2	82.2	<0.001	node 3	
	2668	17	44	67	42	42.1	72.3	43.3	71.3	<0.001		
	3034	1	24	6	16	15.9	95.8	65.4	69.4	<0.001		[10] ^†^
	5509	5	0	0	20	19.6	96.2	72.4	70.4	<0.001		[26] ^†^

Percentages of bacterial isolates showing each peak are shown (type II isolates, 155; type III isolates, 25; type IV isolates, 33; MSSA isolates, 107). SE, SP, PPV, and NPV were calculated by dividing each SCC*mec* type by the total sample number. SE: sensitivity, SP: specificity, PPV: positive predictive value, NPV: negative predictive value, *P*-value for cross-tabulation with other SCC*mec* type. *Reference paper did not include MRSA strains. ^※^Reference paper used MRSA and MSSA isolates. ^†^Reference paper used only MRSA isolates.

**Table 2:** Simple determination of MRSA-specific peaks and each MSSA-specific peak.

Combined Peaks		Database Set		Test Set	
		MRSA (%)	MSSA (%)	MRSA (%)	MSSA (%)
**4-peak determination:** **one or more peaks at** **m/z 1975, 2410, 3890, and 5541 (+)**		96.2	45.8	75.8	53.5
**4-peak determination with**	2194 (-)	80.8	74.8	65.3	61.6
	2230 (-)	86.9	67.3	63.2	69.8
	2339 (-)	88.3	67.3	67.4	58.1
	2630 (-)	76.1	79.4	62.1	66.3

Sensitivities of simple determination with 5-peak combinations are shown. For the prediction of MRSA, the inclusion of one or more peaks at m/z 1975, 2410, 3890, and 5541 and one MSSA-specific peak, such as m/z 2194, 2230, 2339, and 2630, was evaluated.

**Table 3:** Summary of decision tree results.

	Number of Isolates	SE (%)	SP (%)	PPV (%)	NPV (%)
**Database set**	320	96.5	73.0	90.7	88.5
**Test set**	181	87.6	71.4	78.0	83.3

Result of decision tree analyses (Figure 2) using the database and test sets. SE, SP, PPV, and NPV were calculated by dividing each SCC*mec* type by the total sample number. SE: sensitivity, SP: specificity, PPV: positive predictive value, NPV: negative predictive value.

## Supplementary Materials

The following are available online at https://www.mdpi.com/2076-0817/8/4/214/s1.
