# Supplementary material for: Rapid Discrimination of Methicillin-Resistant Staphylococcus aureus by MALDI-TOF MS
Source: Pathogens. 2019 Nov 1;8(4):214. doi: 10.3390/pathogens8040214 (PMC6963962; doi:10.3390/pathogens8040214)
Supplement: Supplementary file 1 [file pathogens-08-00214-s001.pdf]

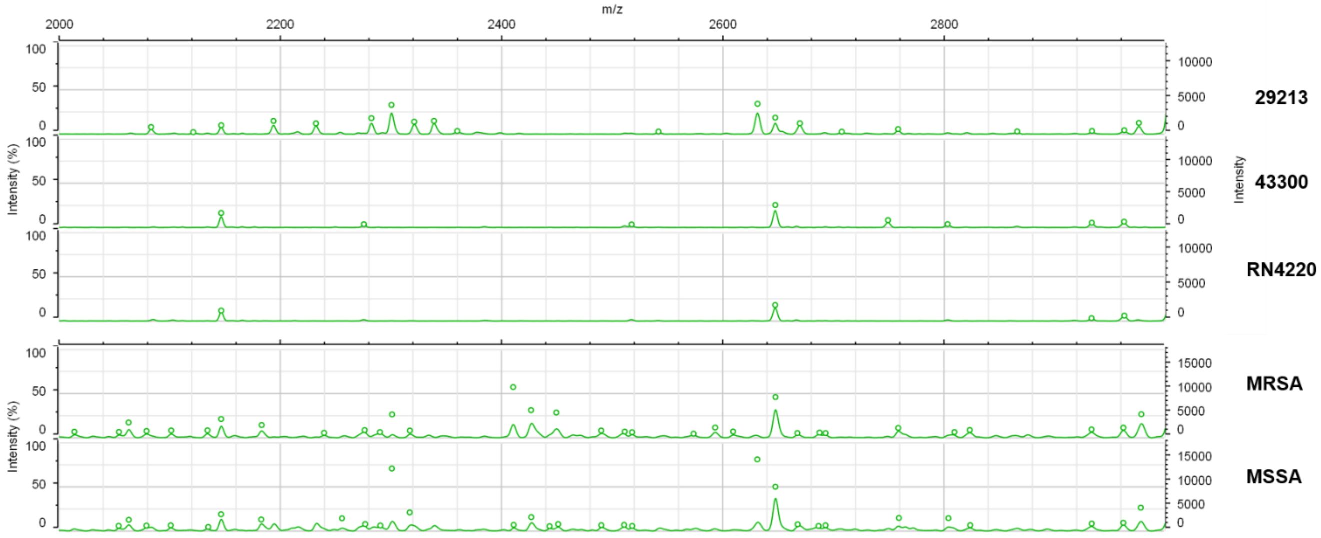

Supplementary Figure S1. Spectra and peak table of ATCC strains. The spectra showed m/z 2000 ~ 4000 range to easily reveal differences in each strain.

Supplementary Table S1. Specific peak of ATCC strains.

	1975	2194	2339	2410	2592	2631	3890	4607	5053	5541	6594	R peak	S peak	Result
ATCC 29213		O	O			O	O		O		O	2	3	grey zone
ATCC 43300									O	O		0	0	no peak
RN4220									O			0	0	no peak

The peak table shows the specific peaks of different strains; O: expressed peak.
